# The Expression of Programmed Death-1 on CD4^+^ and CD8^+^ T Lymphocytes in Patients with Type 2 Diabetes and Severe Sepsis

**DOI:** 10.1371/journal.pone.0159383

**Published:** 2016-07-26

**Authors:** Yumei Jia, Yongzhen Zhao, Chunsheng Li, Rui Shao

**Affiliations:** 1 Department of Endocrinology, Beijing Chaoyang Hospital Affiliated to Capital Medical University, Beijing, China; 2 Department of Emergency Medicine, Beijing Chaoyang Hospital Affiliated to Capital Medical University, Beijing, China; University of Sao Paulo, BRAZIL

## Abstract

**Objective:**

To investigate the expression of Programmed death-1 (PD-1) on T lymphocytes in patients with type 2 diabetes mellitus (T2DM) and severe sepsis, we determined PD-1 expression on CD4^+^ and CD8^+^ T lymphocytes of patients with T2DM, severe sepsis, and T2DM combined with severe sepsis.

**Research Design and Methods:**

This prospective and observational study included 50 healthy controls, 80 cases of T2DM without infection (T2DM group), 88 cases of severe sepsis without T2DM (SS group), and 77 cases of severe sepsis combined with T2DM (SS+T2DM group). Expression of peripheral blood PD-1^+^ CD4^+^ T cells and PD-1^+^ CD8^+^ T cells were compared between these 4 groups. Then, 28-day survival of the SS and SS+T2DM patients was assessed, and the expression of PD-1 on T cells was also compared between survivors and non-survivors.

**Results:**

Percentages of PD-1^+^ CD4^+^ T cells and PD-1^+^ CD8^+^ T cells were higher in the T2DM group than in the healthy control group, and were highest in the SS and SS+T2DM groups. However, the expression of PD-1 on T cells and the mortality showed no significant difference between the SS and SS+T2DM groups. The expression of PD-1 on T cells was higher in non-survivors than survivors, but within the survivor group or non-survivor group, no difference can be detected between those with T2DM and those without T2DM.

**Conclusion:**

The expression of PD-1 on T cells was increased in both T2DM and severe septic patients, but combining T2DM did not cause a further increase on the PD-1 expression in patients with severe sepsis.

## Introduction

Sepsis is a common critical illness in clinical practice, and immunosuppression is an important cause of deterioration of severe sepsis [1]. With the increase in the incidence of type 2 diabetes mellitus (T2DM), its effect on sepsis and the underlying mechanism are attracting more and more attention [2]. T2DM is conventionally considered mostly a metabolic disorder. Insulin resistance and insufficient insulin secretion are considered the core of this disease, which, however, doesn’t well explain the common clinical and biochemical characteristics seen in T2DM patients. In recent years, more and more studies indicate that the pathogenesis of T2DM involves immune dysfunction, and T2DM can be considered somewhat a chronic inflammatory disease [3,4]. Impaired immunity in patients with diabetes may increase the risk of infection, however, the exact effect of diabetes on patients with severe sepsis remains to be studied.

Programmed death-1 (PD-1) is a novel member of the CD28 superfamily. It’s a negative co-stimulatory factor that mediates negative co-stimulatory signal [5]. PD-1 can effectively inhibit functioning and proliferation of the T cells, and reduce IL-2, IL-10 and IFN-γ secretion [6,7], playing an important role in immune regulation [8]. Studies have shown that, PD-1 expression is increased in septic patients, and is closely related to hospital infection and mortality [9,10]. Such effect may be attributed to the immunosuppression effect of PD-1 [11]. Studies about the role of PD-1 on diabetes mostly refer to type 1 diabetes mellitus (T1DM) [12–14], but the role of PD-1 on T2DM has rarely been reported. Furthermore, no study has reported the expression of PD-1 on T cells in type 2 diabetic patients with severe sepsis. This study assessed the expression of PD-1 on the surface of peripheral blood T lymphocytes in healthy volunteers, T2DM patients, severe septic patients, and patients with both T2DM and severe sepsis, to investigate the expression PD-1 on T lymphocytes in patients with T2DM and severe sepsis, and determine the effect of T2DM on the expression of PD-1 in the immune system of patients with severe sepsis.

## Materials and Methods

### Subjects

This observational clinical study was conducted in Beijing Chao-Yang Hospital, a tertiary care teaching hospital, between August 2014 and July 2015. Severe sepsis combined with T2DM (the SS+T2DM group) and severe sepsis without T2DM (the SS group) were enrolled from the emergency department. Meanwhile, infection-free T2DM patients (the T2DM group) who shared a similar history of diabetes and were taking similar drugs as the SS+T2DM group, were selected from the department of endocrinology. In addition, healthy volunteers were recruited as control. Age and gender were matched among the 4 groups. It was required that patients of the T2DM group and the healthy control group should have shown no systemic or local inflammation and had no anti-inflammation drugs in the past week before examination. The diagnosis of diabetes was based on previous hospital records as well as the examination of this time, and the diagnosis of T2DM was consistent with the criteria for diagnosis and classification of diabetes mellitus issued by the American Diabetes Association in 2013 [15]. The diagnosis of severe sepsis was in line with the diagnostic criteria listed in the 2012 International Guidelines for Management of Severe Sepsis and Septic Shock [16]. The exclusion criteria were: age < 18 years, surgical trauma, hematological disease, autoimmune disease, HIV/AIDS, disease of the liver (such as hepatitis, cirrhosis), end-stage renal disease (requiring dialysis), tumors, pregnancy, receiving hormone treatment. Eligible patients may have previously one or more of the following complications: chronic obstructive pulmonary disease (COPD), cardiovascular disease (coronary heart disease and/or heart failure), chronic kidney disease (not including patients that require dialysis) and cerebrovascular disease. Comparison was first performed among the healthy control group, the T2DM group, the SS group, and the SS+T2DM group. Then all patients with severe sepsis (with or without T2DM) were followed up for 28 days, and 28-day mortality of these patients was recorded. The patients who were discharged earlier than 28 days were assessed by telephone to define outcomes. And PD-1 expression was compared between survivors and non-survivors. This study was in line with medical ethics standards, and was approved by the Ethics Committee of Beijing Chaoyang Hospital, and was performed with informed consent from the patients or their families.

### Data Collection

Age, gender, address, telephone number, vital signs, past medical history, treatment for diabetes prior to admission, blood routine examination, blood gas analysis, liver and renal function, hemoglobin A1c (HbA1c), blood glucose levels on admission, high sensitivity C-reactive protein (Hs-CRP), chest X-ray, and results of sputum, blood or urine culture were recorded for all subjects included. HbA1c was measured by Tosoh HLC-723 G8 automatic glycohemoglobin analyzer (Tosoh Corporation, Tokyo, Japan) with the principle of high-performance liquid chromatography. Blood glucose was detected on Siemens ADVIA 2400 automatic biochemistry analyzer (Siemens Diagnostics, Tarrytown, NY, USA) by using glucose oxidase method. Hs-CRP was analyzed by immunoturbidimetric assay (Orion Diagnostica Oy, Espoo, Finland) on the Beckman DXC800 analyzer (Beckman Coulter Inc., Brea, CA, USA). 4ml ulnar vein blood of each subject was taken upon admission and stored in ethylenediamine tetraaccetic acid (EDTA) anticoagulant tubes for flow cytometry analysis. The Acute Physiology and Chronic Health Evaluation System II (APACHE II) score and the Sequential Organ Failure Assessment (SOFA) score were determined within 24 hours.

### Flow Cytometry

**Fig 1:**
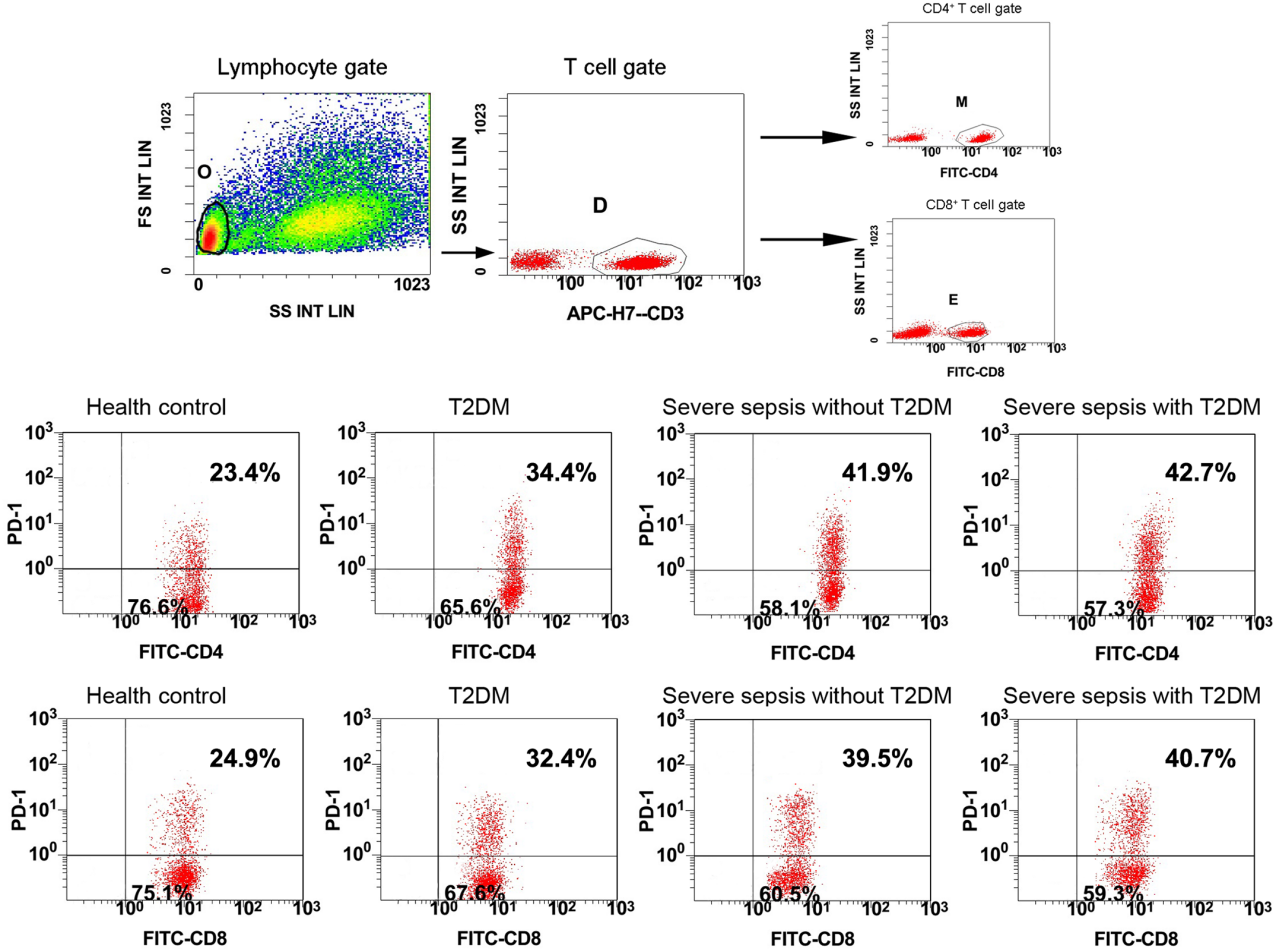
Flow dot plots of gating strategy and the percentages of PD-1 in four groups. All lymphocytes (gate O) were gated by forward scatter/side scatter (FS/SS), differentiating T cell subsets (gate D) by CD3^+^ staining in lymphocytes, and then CD4^+^ T cells (gate M) were further identified based on CD4^+^ staining in T cells, meanwhile, CD8^+^ T cells (gate E) were further identified by CD8^+^ staining in T cells. The multiple dot plots of the percentage of PD-1^+^ CD4^+^ T cells and PD-1^+^ CD8^+^ T cells are shown in four groups: health control, T2DM, severe sepsis without T2DM, and severe sepsis with T2DM. PD-1, programmed death-1; T2DM, Type 2 diabetes mellitus; FITC, fluorescein isothiocyanate; FS INT LIN, forward scatter integral linear; SS INT LIN, side scatter integral linear.

The cells were stained and erythrocytes were lysed by researchers who were blinded to the clinical data. According to the manufacturer’s recommendations, monoclonal antibodies and their isotype controls were used: BV421-labeled anti-PD-1 (5 μl, clone EH12.1; BD Bioscience, San Jose, CA, USA), APC-H7 labeled anti-CD3 (5 μl, clone SK7; BD Bioscience), FITC-labeled anti-CD4 (5 μl, clone OKT4; eBioscience, San Diego, CA, USA), FITC-labeled anti-CD8 (20μl, clone RPA-T8; BD Bioscience) per 100μl of whole blood. Samples were measured on the Gallios^™^ Flow Cytometer (Beckman Coulter, Inc.) and analyzed by Gallios Software Version 1.0 (Beckman Coulter, Inc.). Lymphocytes were gated by forward scatter and side scatter, and T cells subsets were further distinguished by CD3^+^ and CD4^+^ staining, or CD3^+^ and CD8^+^ staining. At least 3,000 CD4^+^ T cells or CD8^+^ T cells were analyzed from each sample. Results are expressed as percentage of PD-1^+^CD4^+^ and PD-1^+^CD8^+^ T cells (Fig 1)

### Statistical Analysis

All data were analyzed using the SPSS17.0 software (SPSS Inc., Chicago, IL, USA). Age, scores, and each examination results were presented as medians with interquartile ranges [M(Q_L_, Q_U_)], and the Mann-Whitney U test was applied; Categorical clinical variables were assessed by chi-square test; correlation between the factors were analyzed by Spearman correlation test; P<0.05 was considered statistically significant.

## Results

### Characteristics of the Patients

A total of 245 patients who met the eligibility criteria and 50 healthy controls were enrolled in this study. There were 80 cases in the T2DM group, 88 cases in the SS group, and 77 cases in the SS+T2DM group. 29 patients failed to survive in the SS group, and 24 patients failed to survive in the SS+T2DM group. Patients of the T2DM group showed a higher level of Hs-CRP, HbA1c, blood glucose levels on admission, and lower lymphocyte count than the healthy controls. The SS and SS+T2DM group showed higher white blood cell count, and Hs-CRP than the healthy control and T2DM group, and lymphocyte count further decreased. The SS+T2DM group had higher HbA1c and blood glucose levels on admission than the T2DM group, but no significant difference was detected in white blood cell count, lymphocyte count, Hs-CRP, APACH II score, SOFA score, and 28-day mortality between the SS and SS+T2DM groups. The SS and SS+T2DM groups showed higher rate of COPD complication than the T2DM group, but rates of other complications showed no significant difference among the 3 groups (Table 1).

**Table 1:** Characteristics of the four groups of patients.

Characteristics	Healthy control (n = 50)	T2DM (n = 80)	Severe sepsis without T2DM (n = 88)	Severe sepsis with T2DM (n = 77)
Male, n (%)	27(54.0)	43(53.8)	46(52.3)	41(53.2)
Age, years	61(52–71)	61(50–72)	62(53–71)	64(53–71)
WBC (×10^9^/L)	6.67(5.89–7.73)	6.81(6.06–8.10)	14.10(11.80–17.35) *^#^	13.50(10.52–16.78) *^#^
Lymphocytes (×10^9^/L)	2.68(2.32–3.05)	2.37(1.92–2.93) *	0.87(0.64–1.41) *^#^	0.90(0.50–1.55) *^#^
Hs-CRP (mg/L)	0.50(0.24–1.02)	1.94(1.30–3.93) *	12.96(10.65–14.39) *^#^	13.08(11.13–14.58) *^#^
HbA1c (%)	5.70(5.23–6.05)	9.55(8.80–11.10) *^△^	5.73(5.22–6.10)	11.30(8.65–13.30)*^#^^△^
Glucose levels (mmol/L)	5.23(4.81–5.64)	8.33(6.68–10.28)*	7.11(6.10–9.23)*	11.80(7.30–16.25)*^#^^△^
APACHE II score	/	/	19(15–25)	20(16–23)
SOFA score	/	/	11(8–14)	11(8–15)
Percentage of PD-1^+^CD4^+^ T cells (%)	25.98(22.38–29.15)	32.78(27.01–38.16) *	39.60(31.42–48.02)*^#^	40.34(30.79–46.13)*^#^
Percentage of PD-1^+^CD8^+^ T cells (%)	21.48(16.70–25.39)	32.90(23.89–40.51)*	38.72(29.84–51.59)*^#^	41.02(31.55–48.55)*^#^
MFI of PD-1^+^CD4^+^ T cells	1.32(1.21–1.85)	4.54(3.70–4.83)*	5.78(3.68–8.12)*^#^	5.67(4.51–7.30)*^#^
MFI of PD-1^+^CD8^+^ T cells	1.36(0.97–2.55)	4.94(4.15–5.57)*	5.69(4.29–8.13)*^#^	5.77(4.40–7.52)*^#^
Comorbidities, n (%)				
COPD	/	16(20.0)	36(40.9) ^#^	29(37.7) ^#^
Cardiovascular disease	/	30(37.5)	34(38.6)	31(40.3)
Chronic renal disease	/	19(23.8)	18(20.5)	21(27.3)
Cerebrovascular disease	/	6(7.5)	13(14.8)	12(15.6)
Mortality, *n* (%)	/	/	29(33.0)	24(31.2)

T2DM, Type 2 diabetes mellitus; WBC, white blood cells; Hs-CRP, high sensitivity C-reactive protein; HbA1c, hemoglobin A1c; APACHE II, acute physiology and chronic health evaluation system II; SOFA, sequential organ failure assessment; COPD, chronic obstructive pulmonary disease; PD-1, programmed death-1; MFI, mean of fluorescence intensity.

* compared to the Health control group *P*<0.05;

^#^ compared to the T2DM group *P*<0.05;

^△^ compared to the Severe sepsis without T2DM group *P*<0.05.

### The Expression of PD-1^+^ CD4^+^ T Cells and PD-1^+^ CD8^+^ T Cells

Percentages of PD-1^+^ CD4^+^ T cells and PD-1^+^ CD8^+^ T cells were significantly increased in the T2DM, SS, and SS+T2DM groups than in the healthy control group, and were the highest in the SS+T2DM and SS groups, but no significant difference was detected between the SS+T2DM group and SS group (Fig 1, Table 1). Similar results were shown when flow cytometry data were expressed as mean fluorescence intensity (MFI) (Table 1).

### The Correlation between the Expression of PD-1 on T Cells and the Severity of Illness

**Fig 2:**
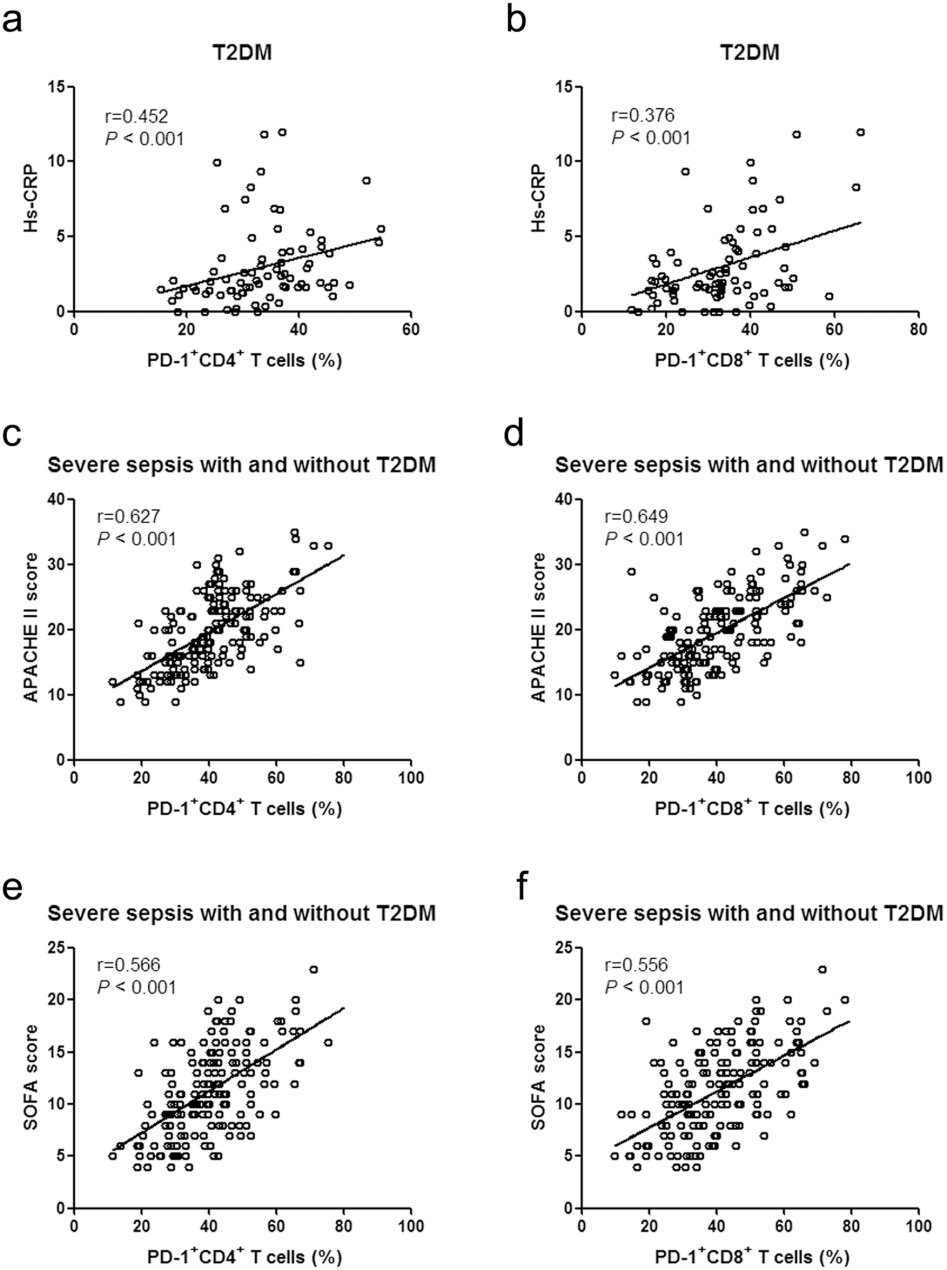
Correlation of the percentage of PD-1^+^ T cells with Hs-CRP and the score system. (a) Correlation of Hs-CRP and the percentage of PD-1^+^CD4^+^ T cells in the patients with T2DM. (b) Correlation of Hs-CRP and the percentage of PD-1^+^CD8^+^ T cells in the patients with T2DM. (c) Correlation of the APACHE II score and the percentage of PD-1^+^CD4^+^ T cells in the patients with severe sepsis (with and without T2DM). (d) Correlation of the APACHE II score and the percentage of PD-1^+^CD8^+^ T cells in the patients with severe sepsis (with and without T2DM). (e) Correlation of the SOFA score and the percentage of PD-1^+^CD4^+^ T cells in the patients with severe sepsis (with and without T2DM). (f) Correlation of the SOFA score and the percentage of PD-1^+^CD8^+^ T cells in the patients with severe sepsis (with and without T2DM). PD-1, programmed death-1; T2DM, Type 2 diabetes mellitus; Hs-CRP, high sensitivity C-reactive protein; APACHE II, acute physiology and chronic health evaluation system II; SOFA, sequential organ failure assessment.

For patient of the T2DM group, percentages of PD-1^+^ CD4^+^ T cells and PD-1^+^ CD8^+^ T cells were both positively correlated with Hs-CRP levels (r = 0.452 and 0.376 respectively, P<0.001 for both) (Fig 2a and 2b), but showed no significant correlation with HbA1c, blood glucose levels on admission (P>0.05). For patients with severe sepsis (with and without T2DM), the percentages of PD-1^+^ CD4^+^ T cells and PD-1^+^ CD8^+^ T cells were positively correlated with APACHE II score (r = 0.627 and 0.649 respectively, P<0.001) or with SOFA score (r = 0.566 and 0.556 respectively, P<0.001) (Fig 2c–2f), but showed no significant correlation with Hs-CRP, HbA1c, blood glucose levels on admission or lymphocyte count (P>0.05). Positive correlations were also observed when MFI of PD-1^+^ CD4^+^ T cells and PD-1^+^ CD8^+^ T cells were correlated with APACHE II score (r = 0.345 and 0.245 respectively, P<0.01) or with SOFA score (r = 0.376 and 0.320 respectively, P<0.001).

### Comparison between the Survivor Group and Non-Survivor Group

**Fig 3:**
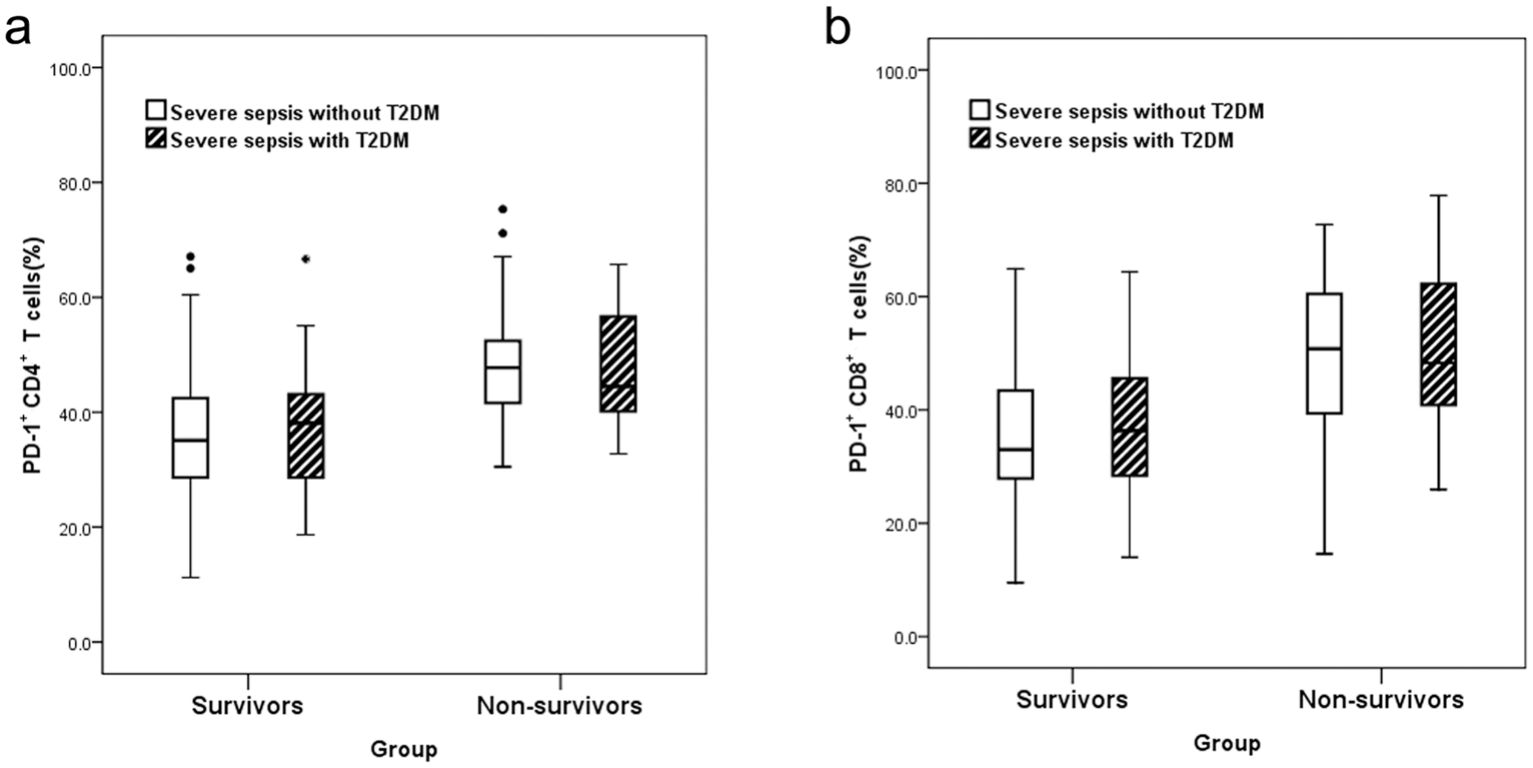
Comparison between non-survivors and survivors among severe septic patients with or without T2DM. (a) Percentage of PD-1^+^ CD4^+^ T cells. (b) Percentage of PD-1^+^ CD8^+^ T cells. The percentage of PD-1 on T cells was higher in non-survivors than survivors (P<0.05), but within the survivor group or non-survivor group, no difference can be detected between those with T2DM and those without T2DM (P>0.05). The dot symbols in Fig 3A are outliers, but they do not represent any statistical significance. PD-1, programmed death-1; T2DM, Type 2 diabetes mellitus.

Percentages of PD-1^+^CD4^+^ T cells and PD-1^+^CD8^+^ T cells, MFI of PD-1^+^ CD4^+^ T cells and PD-1^+^ CD8^+^ T cells, APACH II score and SOFA score were significantly higher in non-survivors than survivors among severe septic patients with or without T2DM (P<0.05), the non-survivors of the SS+T2DM group were older than survivors (P<0.05), but within the survivor group or non-survivor group, no difference could be detected between those with T2DM and those without T2DM (P>0.05) (Table 2, Fig 3). Gender, white blood cell count, lymphocyte count, Hs-CRP, HbA1c, blood glucose levels on admission, as well as the site of infection all showed no significant difference between the survivor group and non-survivor group (P>0.05).

**Table 2:** Comparison between the survivors and the non-survivors.

	Severe sepsis without T2DM			Severe sepsis with T2DM		
Characteristics	Survivors (n = 59)	Non-survivors (n = 29)	*p*^a^	Survivors (n = 53)	Non-survivors (n = 24)	*p*^b^
Male, n (%)	32(54.2)	14(48.3)	0.601	30(56.6)	11(45.8)	0.383
Age, years	61(50–70)	66(59–71)	0.053	63(45–70)	67(59–74)	0.028
WBC (×10^9^/L)	14.90(12.70–17.20)	11.80(10.30–17.55)	0.063	13.37(10.21–16.78)	14.38(11.57–17.50)	0.538
Lymphocytes (×10^9^/L)	0.86(0.65–1.35)	0.87(0.55–1.63)	0.793	0.90(0.45–1.45)	1.05(0.55–1.58)	0.640
Hs-CRP (mg/L)	12.88(10.68–14.21)	13.15(9.90–14.96)	0.570	13.08(10.68–14.47)	13.05(11.33–14.88)	0.779
HbA1c (%)	5.72(5.00–6.11)	5.74(5.39–6.06)	0.716	11.20(8.70–13.20)	11.65(8.63–13.50)	0.700
Glucose levels (mmol/L)	7.00(6.11–9.16)	7.25(6.00–9.83)	0.526	12.20(7.60–16.85)	11.40(5.28–16.13)	0.269
APACHE II score	17(13–22)	25(21–28)	<0.001	18(14–21)	25(20–27)	<0.001
SOFA score	10(7–13)	14(9–16)	0.001	10(7–13)	14(12–18)	<0.001
Percentage of PD-1^+^CD4^+^ T cells (%)	35.10(28.62–42.93)	47.76(40.19–51.73)	<0.001	38.12(28.85–43.12)	44.50(40.16–56.90)	<0.001
Percentage of PD-1^+^CD8^+^ T cells (%)	32.99(27.90–43.80)	50.80(38.75–61.10)	<0.001	36.34(27.77–45.68)	48.28(40.72–62.87)	<0.001
MFI of PD-1^+^CD4^+^ T cells	4.98(3.26–7.01)	7.29(5.61–9.14)	0.002	5.43(4.29–6.33)	6.92(5.46–7.80)	0.011
MFI of PD-1^+^CD8^+^ T cells	5.24(4.05–7.71)	7.45(4.99–9.43)	0.030	5.50(4.18–6.32)	7.12(5.35–8.37)	0.015
Primary site of infection, n (%)						
Respiratory	32(54.2)	21(72.4)	0.102	28(54.2)	17(70.8)	0.138
Abdominal	12(20.3)	6(20.7)	0.969	12(22.6)	4(16.6)	0.768
Urinary	8(13.6)	2(6.9)	0.570	7(13.2)	1(4.2)	0.423
Cerebral	3(5.1)	0	/	3(5.7)	0	/
Others	4(6.8)	0	/	3(5.7)	2(8.3)	1.000

T2DM, Type 2 diabetes mellitus; WBC, white blood cells; Hs-CRP, high sensitivity C-reactive protein; HbA1c, hemoglobin A1c; APACHE II, acute physiology and chronic health evaluation system II; SOFA, sequential organ failure assessment; PD-1, programmed death-1; MFI, mean of fluorescence intensity.

^a^ The survivors compared with the non-survivors in the patients of Severe sepsis without T2DM.

^b^ The survivors compared with the non-survivors in the patients of Severe sepsis with T2DM.

## Discussion

PD-1 is a negative co-stimulatory molecule that negatively regulates the immune system. PD-1 transduces an inhibitory signal that leads to inhibition of activation, proliferation, and functioning of the effector cells [7]. In this study, it was found that PD-1 levels on peripheral blood T lymphocytes was increased in both the T2DM patients and those with severe sepsis compared to healthy controls, and those with severe sepsis showed higher PD-1 levels than T2DM, suggesting that both T2DM patients and those with severe sepsis had immune suppression. However, no additive effect was observed when T2DM and severe sepsis were combined, patients with severe sepsis combining T2DM had similar expression of PD-1^+^ CD4^+^ T cells and PD-1^+^ CD8^+^ T cells, and similar mortality rate as those with severe sepsis only. Our results suggest that T2DM did not increase 28-day mortality in patients with severe sepsis, nor did it increase the expression of PD-1 on T cells.

More and more studies suggest that T2DM is an immune-related chronic inflammatory disease, and when it affects the islet, decreased number of β cells and insulin secretion disorder may occur [17]. T2DM patients and animal model of T2DM both showed immune cell invasion of the islet [18]. It was found in this study that expressions of peripheral blood PD-1^+^ CD4^+^ T cells and PD-1^+^ CD8^+^ T cells were increased in the T2DM patients compared with healthy control, and were positively correlated with Hs-CRP level. Hs-CRP as a marker of inflammation has been widely used in clinical research. It has been reported that increased Hs-CRP is of significant diagnostic value for the severity of diabetes, diabetic nephropathy, and diabetic coronary heart disease [19–21]. So, the expression of PD-1 on T cells could be correlated with the severity of diabetes. It has also been reported that PD-1 levels are increased on CD4^+^ CD28^-^ T cells of T2DM patients, which are closely correlated with the severity of diabetic atherosclerotic macrovascular diseases [22].

The one of the major causes of death in patients with severe sepsis is immune suppression caused by damages to the immune system [1, 23], in which the negative co-stimulatory molecules such as PD-1 may play a major role [11]. Autopsy results demonstrated that PD-1 levels were significantly increased on the surface of lymphocytes in the spleen of the deceased [1]. Animal experiments also demonstrated higher PD-1 expression on the surface of peripheral blood macrophages and monocytes in the mouse model of sepsis [24], and administration of PD-1 antagonist significantly improved survival of these mice [25]. Recent clinical trials also indicate that PD-1 expression on peripheral blood T cells is significantly increased in patients with sepsis [9, 10, 26]. Such increase in PD-1 expression on T cells was also obvious in patients with severe sepsis as included in this study, and PD-1 levels were positively correlated with APACHE II and SOFA scores, suggesting that PD-1 levels were closely related with severity of the disease. This study found that PD-1 levels on T cells were higher in the non-survivors, who might be associated with more severe immunosuppression. The expression of PD-1 on T cells showed no significant difference between the SS group and the SS+T2DM group, possibly because T2DM-related PD-1 increase was not so obvious as that caused by severe sepsis, it’s only slightly increased compared with healthy controls. Animal experiment demonstrated that PD-1 has a protective effect on T1DM. Injecting PD-1 antibody into nonobese diabetic (NOD) mice model of diabetes accelerated occurrence of diabetes, and it also plays a role in islet inflammation and production of T-cell pro-inflammatory cytokines [12]. Lack of the PD-1 inhibitory signal could lead to infiltration of CD8^+^ T cells into islet, where they destroy islet β cells and release inflammatory cytokines [27]. Similarly, reports on PD-1 and T2DM also indicate that the increase of PD-1 levels in the mice model of T2DM nephropathy was a compensatory response to reduce inflammation [28]. So, it is suspected that PD-1 levels increase in DM may represent some compensatory self-protection mechanism, while the marked increase of PD-1 levels in severe sepsis may represent excessive activation of this negative regulation mechanism, which results in adverse consequences. Because the effect of PD-1 on T cells in T2DM and severe sepsis is different, the increase of PD-1 on T cells in T2DM and severe sepsis can not be simply added to each other, and combining T2DM do not cause a further increase on the expression of PD-1 in patients with severe sepsis.

It has been reported that diabetes may increase the risk of infection and sepsis [29, 30]. In this study, blood glucose levels on admission and HbA1c of the SS+T2DM group were higher than that of the T2DM group, suggesting that poor glycemic control may be one of the causes for severe infection among patients with T2DM. Although diabetes seems to be a risk factor for infection, its effect on the prognosis of patients with sepsis remains controversial [31]. In a retrospective cohort study on patients with severe sepsis admitted to ICU, it was found that those with T2DM showed no significant differences with those without, as to number of organ dysfunctions, length of hospital stay, and 90-day hospital mortality [32]. In this study, APACHE II score and SOFA score also showed no significant difference between the SS group and SS+T2DM group, which meant that T2DM did not aggravate the conditions of patients with severe sepsis. Many researches indicated that the incidence of sepsis and the risk of mortality were increased in the elderly patients [33, 34], our study also found the non-survivors of the SS+T2DM group were older than survivors. But within the survivor group or non-survivor group, age and the expression of PD-1 on T cells all showed no difference between severe sepsis with T2DM and severe sepsis without T2DM. PD-1 levels showed no significant difference between the SS group and the SS+T2DM group, suggesting that diabetes did not cause further immunosuppression in patients with severe sepsis, and whether diabetic immunosuppression represents a risk factor for infection and how it affects progression of severe sepsis remain to be clarified.

This study also has some limitations. First, in order to demonstrate the effect of T2DM on immunity of patients with severe sepsis, this study selected infection-free T2DM patients that had a similar history of diabetes and similar medication as those patients with both T2DM and severe sepsis, however, the effect of some confounding factors, such as metabolic control, diabetes-related complications, obesity, and insulin or other medications, on the prognosis of severe sepsis with diabetes can not be ruled out [35]. Second, we did not calculate the absolute numbers of PD-1^+^ CD4^+^ and PD-1^+^ CD8^+^ T cells, the absolute numbers of PD-1^+^ CD4^+^ and PD-1^+^ CD8^+^ T cells may provide more precise information. Third, all patients with severe sepsis included in this study were selected from the emergency department, and the blood sample was taken only upon admission and without continuous monitoring; in addition, this study was performed in a single center, so the conclusion should be verified by future large-scale multi-center studies. At last, this is only an observational study, and the underlying molecular mechanism remains to be clarified.

## Conclusions

The PD-1 levels were increased in peripheral blood CD4^+^ T cells and CD8^+^ T cells of both T2DM patients and those with severe sepsis, suggesting that both diseases involve immune suppression. However, combining T2DM did not cause a further increase in PD-1 levels among patients with severe sepsis, suggesting that diabetes did not cause further immune suppression, so whether diabetic immunosuppression represents a risk factor for infection and how it affect progression of severe sepsis remains to be investigated.

## Supporting Information

S1 Checklist SROBE checklist. (DOC)

S1 Data Raw data. (XLS)
